# Reducing alcohol and/or cocaine-induced reward and toxicity via an epidermal stem cell-based gene delivery platform

**DOI:** 10.1038/s41380-021-01043-y

**Published:** 2021-02-22

**Authors:** Qingyao Kong, Yuanyuan Li, Jiping Yue, Xiaoyang Wu, Ming Xu

**Affiliations:** 1 Department of Anesthesia and Critical Care, The University of Chicago, Chicago, IL USA; 2 Ben May Department for Cancer Research, The University of Chicago, Chicago, IL USA

**Keywords:** Stem cells, Addiction

## Abstract

Alcohol use disorder (AUD) is one of the foremost public health problems. Alcohol is also frequently co-abused with cocaine. There is a huge unmet need for the treatment of AUD and/or cocaine co-abuse. We recently demonstrated that skin grafts generated from mouse epidermal stem cells that had been engineered by CRISPR-mediated genome editing could be transplanted onto mice as a gene delivery platform. Here, we show that expression of the glucagon-like peptide-1 (GLP1) gene delivered by epidermal stem cells attenuated development and reinstatement of alcohol-induced drug-taking and seeking as well as voluntary oral alcohol consumption. GLP1 derived from the skin grafts decreased alcohol-induced increase in dopamine levels in the nucleus accumbens. In exploring the potential of this platform in reducing concurrent use of drugs, we developed a novel co-grafting procedure for both modified human butyrylcholinesterase (hBChE)- and GLP1-expressing cells. Epidermal stem cell-derived hBChE and GLP1 reduced acquisition of drug-taking and toxicity induced by alcohol and cocaine co-administration. These results imply that cutaneous gene delivery through skin transplants may add a new option to treat drug abuse and co-abuse.

## Introduction

Alcohol use disorder (AUD) is a chronic brain disease and one of the foremost public health problems [1]. AUD is one of the most prevalent psychiatric disorders worldwide and its lifetime prevalence is 29.1% in the United States [2]. AUD is also characterized by high comorbidity. The lifetime prevalence of occurrence of comorbid alcoholism in cocaine abusers is 60–85% [3, 4]. Concurrent use of alcohol and cocaine produces cocaethylene which inhibits the dopamine (DA) transporter and produces euphoria [5], and it has cardiotoxic effects by blocking cellular sodium channels with a potency that is equal to or greater than cocaine [6, 7]. Cocaethylene has a 3–5 times longer half-life in the plasma than cocaine and the LD50 of cocaethylene is substantially lower than cocaine. Consequently, there is a 18–25 fold higher risk of death than using cocaine alone [8]. The peak concentration of cocaine is also increased by roughly 20% in the presence of alcohol, likely due to a higher rate of absorption. The higher cocaine level in the blood in the presence of alcohol is also linked to higher cardiotoxicity induced by cocaine [8, 9]. There is a huge unmet need for the treatment of AUD and/or cocaine co-abuse.

Glucagon-like peptide 1 (GLP1) is a major physiological incretin that is expressed by the intestinal epithelial endocrine L-cells and neurons in the nucleus of the solitary tract, and it circulates in the blood to control food intake and glucose homeostasis [10]. GLP1 receptors (GLP1R) are also expressed in brain reward areas including the ventral tegmental area (VTA) and nucleus accumbens (NAc). Natural rewards can induce DA elevation in the NAc [11, 12], and GLP1 or GLP1 receptor agonists can reduce palatable food intake by suppressing mesolimbic DA signaling or DA responses to food-predictive cues, respectively [13, 14]. Emerging evidence suggests that GLP1, GLP1R agonists, and antagonists can modulate drug reward behaviors including those induced by alcohol and cocaine [15–20]. Moreover, GLP1 acts via GLP1R on the mesolimbic DA system to attenuate the neurobiological effects of drugs of abuse including reward behaviors and DA release in the NAc in mice [19, 20]. Butyrylcholinesterase (BChE) is an endogenous enzyme that hydrolyzes its normal substrate acetylcholine [21]. BChE is expressed by hepatocytes and it circulates in the blood. Advances in computer-directed protein engineering have significantly increased the catalytic activity of the human BChE (hBChE) for degrading cocaine [22, 23]. This designed enzyme can also metabolize cocaethylene. Both GLP1 and hBChE have very short half-lives in vivo [24, 25], however, and the use of GLP1 receptor agonists requires long-term and parenteral administration which may be inconvenient and costly for patients, limiting their potential in treating alcohol and/or cocaine abuse.

We previously developed an epidermal stem cell-based long-term gene delivery platform that uses CRISPR/CAS9 technology to genetically engineer mouse epidermal stem cells and grafting these modified cells to normal mouse donors. We found that inducible expression of GLP1 from the skin was able to reverse diet-induced obesity and diabetes [26]. Moreover, engineered skin grafts producing hBChE were effective in metabolizing cocaine in circulation at a fast rate, decreasing DA levels in the brain and protecting mice from cocaine-seeking and overdose [27]. In the present study, we utilized this gene delivery platform to examine whether skin-derived GLP1 can reduce alcohol reward and active ongoing consumption. To explore the potential of this platform in reducing concurrent drug use, we developed a novel co-culture and co-grafting procedure to allow simultaneous expression of both hBChE and GLP1. We found that cutaneous expression of the GLP1 gene attenuated development and reinstatement of alcohol-induced drug-taking and seeking as well as voluntary oral alcohol consumption. Moreover, skin cell-derived hBChE and GLP1 reduced acquisition of drug-taking and toxicity induced by alcohol and cocaine co-administration. These results imply that cutaneous gene delivery through skin transplants may add a new option to drug abuse and co-abuse.

## Materials and methods

### Mice

CD1 male adult mice (30–40 g) were used for all behavioral and neurochemical experiments. Animals were group-housed in a specific pathogen-free facility under standardized conditions on a 12-h light/dark cycle with water and food freely available as described before [27]. All experimental procedures were approved by the IACUC of the University and were in accordance with the *Guide for the Care and Use of Laboratory Animal* (8th edition).

### Drugs and reagents

Ethanol (Sigma-Aldrich, Saint Louis, MO, 95%, density = 0.816) for intraperitoneally (i.p.) injections was prepared in sterile saline at different concentrations (10 ml/kg). Sterile saline was injected i.p. at 10 ml/kg. Alcohol was prepared at 3%, 6%, and 9% (v/v%) in acidified drinking water. Saccharin (Sigma-Aldrich, Saint Louis, MO) was prepared at 0.06% and 0.12% (w/v%) in acidified drinking water. Cocaine HCl (Sigma-Aldrich, Saint Louis, MO) was prepared using sterile saline and injected i.p. (10 ml/kg) at different concentrations. Antibodies and mouse cell lines utilized in the present study were described previously [26, 27].

### Generating GhBChE/GLP1 co-grafted mice

We previously made genetically modified skin stem cells carrying either GLP1 or hBChE [26, 27]. Mice were randomly allocated to different groups that received engraftment with GLP1 (GGLP1 group), both hBChE and GLP1 (GhBChE/GLP1 group), or mock controls (GWT). Circular shaped decellularized dermis (1 cm diameter) was prepared by treating newborn mouse skins using EDTA. In total, 7.5 × 10^5^ each GLP1 and hBChE expressing keratinocytes were plated onto the mouse dermis [26]. The skin culture was used to graft CD1 male recipient mice at 6–8 weeks of age with the help of a silicone chamber bottom. The chamber cover was opened to allow the graft to get exposed to the air after 7 days. Anti-CD4 (GK1.5, 0.2 mg) and anti-CD8 (2.43.1, 0.2 mg) antibodies were injected i.p. for skin grafting.

### Conditioned place preference (CPP) procedure

CPP procedures were modified from our previous methods [27]. All CPP behavioral boxes were purchased from (Med Associates, E. Fairfield, VT, USA). These boxes consist of two large chambers separated by a small chamber as described before. The two large chambers have different combinations of color, visual, and floor texture properties. A biased design was used for all CPP studies.

In alcohol-related studies, after 7–10 days of recovery from graft surgery, mice went through acquisition, extinction, and reinstatement evaluations, respectively. CPP acquisition included pretest, conditioning, and expression test. In pretest on day 1, mice that showed an obvious bias (larger than 500 s in the middle chamber or bigger than 800 s in either large chamber) were not used based on pre-established criteria. During conditioning phase days 2–5, mice were given i.p. injections of alcohol (1 g/kg) and saline at an interval of 5 h daily. Immediately after alcohol or saline injections, the individual mouse was confined to the white chamber or black chamber for 30 min alternatively. On day 6, CPP expression was conducted by letting mice have access to the entire boxes for 20 min with no drug or saline injections. Extinction training followed the CPP acquisition phase. During this phase, mice were let to have access to the entire box for 20 min with no injections. This extinction training was repeated daily. Mice were considered to have reached extinction when CPP levels reduced to the pretest level in two consecutive days. For reinstatement, mice were given a 0.5 g/kg alcohol injection and were tested for CPP for 20 min. Time in the drug chamber minus time in the vehicle chamber was used for analyzing CPP data for CPP acquisition, extinction, and reinstatement.

For the co-grafting study, 10 days after recovery from skin grafting surgeries, one group each of GhBChE/GLP1 and GWT mice was given a doxycycline diet and trained for CPP acquisition induced by alcohol and cocaine. Mice underwent pretest on day 1, 4 days of alternating drug and vehicle conditioning on days 2–5, and CPP test on day 6.

### Two-bottle choice test

This was performed as described [28]. One group each of GGLP1 and GWT mice were single-housed and fed with a doxycycline diet at the start of 6% ethanol. Mice were presented with two 15 ml inverted graduated bottles with stainless steel water stoppers, one containing acidified water, the other containing 3%, 6%, or 9% alcohol, in their home cage. Regular cages were used whose wire tops were divided into two compartments, the food is evenly placed in both compartments and one bottle is inserted in each compartment. Bottles were inserted so that the tips of the sprouts were at approximately the same distance from the floor of the cage. Mice underwent 4 weeks of two-bottle preference testing. Mice were presented with water vs. water in the first week, 3% alcohol vs. water in the second week, 6% alcohol vs. water in the third week, and 9% alcohol and water in the fourth week. Mice were accessed to two bottles 24 h daily, and bottles were switched and refilled every other day. The alcohol preference ratio was derived by dividing the alcohol solution consumption by the total fluid consumption for every alcohol concentration. One week after the alcohol two-bottle choice procedure, the same GGLP1 and GWT mice were tested for saccharin fluid drinking by providing first 0.06% and 0.12% saccharin solutions both for 1 week. The saccharin fluid consumptions were recorded every other day. Similarly, the saccharin preference ratio was determined at every concentration by dividing the saccharin solution consumption by total fluid consumption.

### Acute drug overdose test

We performed grating surgeries using four groups of GhBChE/GLP1 and four groups of GWT mice and they were allowed to recover for 10–12 days. Mice were given i.p. sequential injections of 60 mg/kg cocaine plus 0.33 g/kg ethanol, 60 mg/kg cocaine plus 2 g/kg ethanol, 60 mg/kg cocaine plus 3 g/kg ethanol, 60 mg/kg cocaine plus 5 g/kg ethanol, respectively. The drug dose ranges used were similar to a published report [29]. We closely observed all mice for 4 h after drug injections and plotted the percent lethality induced by cocaine and alcohol co-administration.

### Microdialysis and extracellular DA measurement

Microdialysis and DA concentration measuring methods are modified from our previous study [27]. In brief, GGLP1 and GWT mice that were on a doxycycline diet were anesthetized and secured in a stereotaxic instrument. A guide cannula was unilaterally implanted, and a microdialysis probe was positioned in the guide cannula and reached the NAc in the mouse that is freely moving. During dialysate sampling, the microdialysis probe was infused with artificial cerebrospinal fluid at a rate of 1 μL/min. Sampling was started 30 min prior to 1 g/kg alcohol i.p. injection. Then, the dialyzed samples were harvested at intervals of 30 min. For measuring extracellular DA levels, benzoyl chloride was added to dialysate samples for derivatization. DA concentration in derivatized dialysate samples was analyzed using liquid chromatography–mass spectrometry (LC–MS). Probe tracks were verified by the histological method after microdialysis.

### Plasma alcohol concentrations

GGLP1 and GWT mice were fed with a doxycycline diet, and plasma alcohol concentrations were determined after an acute i.p. alcohol (1 g/kg) administration following a 4-h session. Totally, 100 μl whole blood was collected from mouse caudal vein 0 min, 30 min, 1 h, 2 h, 4 h after alcohol administration, and serum was extracted at 2000*g*, 5 min. An ethanol assay kit (Abcam, ab65343, Cambridge, UK) was used for quantifying the alcohol levels in the serum samples.

### Plasma cocaethylene measurement using LC–MS

In GhBChE/GLP1 and GWT mice that were on a doxycycline diet, blood samples from the tail vein were collected 0, 15, 30, 60 min after sequential i.p. injections of 60 mg/kg cocaine plus 0.33 g/kg ethanol. Samples were extracted following a modified QuEChERS method [30]. Totally, 100 μl of the blood sample was diluted by three and mixed with extraction preparation (100 mg) and internal standard solution (200 μl, Agilent SampliQ) and spun at 4400 rpm for 5 min. The solid-phase extraction (Agilent SampliQ) was added to the supernatant (~200 µl), and the mixture was spun at 4400 rpm for 1 min. The upper layer was collected, and 10 µl of the sample was used in liquid chromatography–mass spectrometry (LC–MS)/MS for measuring cocaethylene using the methods we previously described [27].

### Statistical analysis

Data were analyzed with Microsoft Excel and GraphPad Prism software. Data distribution and variance were tested using Shapiro–Wilk normality tests. Normally distributed were analyzed with Mixed-model ANOVA or repeated-measures ANOVA to determine the difference between the means of different experimental groups. Significance was tested using the post hoc Fisher’s least significant difference test. The sample size was chosen based on previous studies. No additional statistical methods were used to estimate the sample size. Investigators were blinded to the different group allocations.

## Results

### Cutaneous gene delivery of GLP1 attenuates the development of alcohol-induced drug-taking, seeking, and ongoing alcohol drinking

We previously made a DNA construct containing a human GLP1 gene under the control of a doxycycline promotor and the GGLP1 grafted mice. After 3 days of feeding the mice with a doxycycline-containing diet, GLP1 levels in the blood drastically increased, and GLP1 was steadily expressed in GGLP1 mice for at least 16 weeks [26]. GLP1R activation, either by GLP1 or GLP1R agonists, is also able to attenuate the rewarding effects of alcohol [19, 20]. We used GGLP1 mice and investigated the potential effects of skin-derived GLP1 on alcohol reward using the CPP behavioral paradigm.

**Fig. 1:**
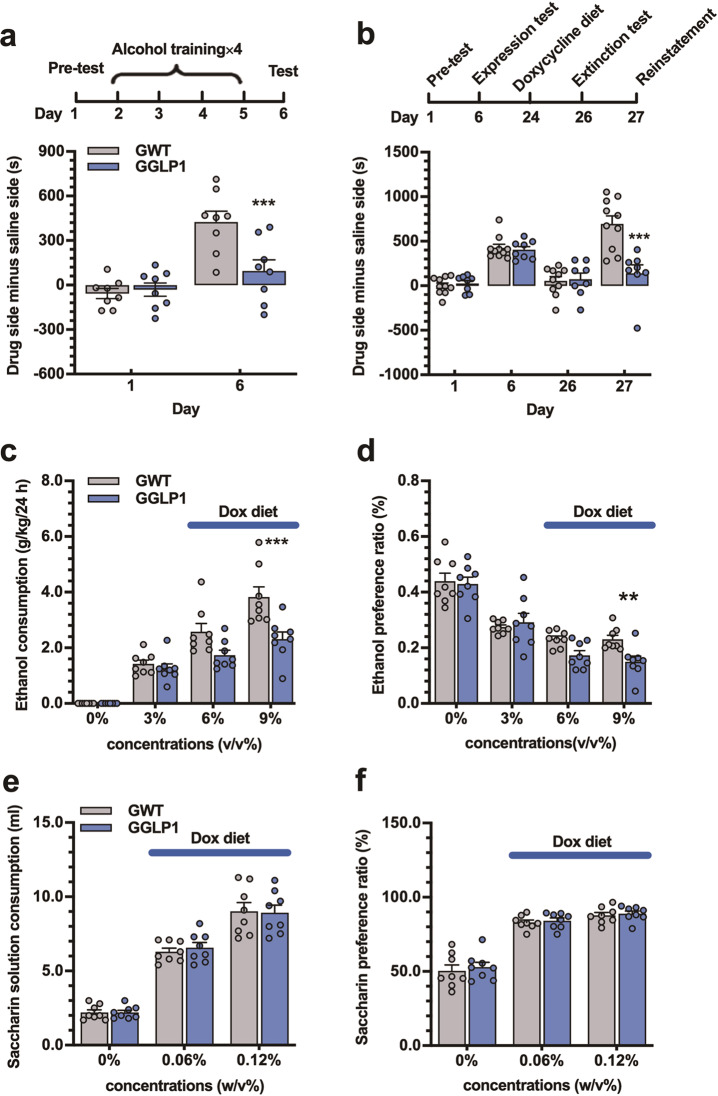
Cutaneous gene delivery of GLP1 decreases the development of alcohol-induced-taking, seeking, and ongoing alcohol drinking. **a** Alcohol-induced acquisition of CPP. GGLP1 and GWT mice were fed with doxycycline and underwent pre-test (day 1), alcohol conditioning (1 g/kg, days 2–5), and expression test (day 6). Data were plotted as means ± S.E.M. with *n* = 8 mice per group. Treatment × days interaction by repeated-measures ANOVA: *F*_1,14_ = 10.79, *P* = 0.0054). Significance level was determined by Fisher’s least significant difference (LSD) test. ***P = 0.0005 on day 6. **b** Alcohol-induced reinstatement of CPP. GGLP1 and control mice underwent extinction procedures (Days 7–26), and a doxycycline diet was fed from day 24. On day 27, GGLP1 and GWT mice received an injection of alcohol (0.5 g/kg), and the CPP level was tested again. (*n* = 8 mice in GGLP1 group, *n* = 10 mice in GWT group; treatment × days interaction by mixed-model ANOVA: *F*_3,48_ = 30.40, *P* < 0.0001). Significance level was tested by Fisher’s LSD test as before. ***P < 0.0001 between GGLP1 and GWT mice on day 27. **c** Alcohol consumption (g/kg/24 h) in GGLP1 and GWT mice were measured at various alcohol concentrations. Bars represent mean ± S.E.M. (*n* = 8 mice per group; treatment × days interaction by repeated-measures ANOVA: *F*_2,28_ = 7.360, *P* = 0.0027). Significance was tested by Fisher’s LSD test. *P = 0.0321 and **P = 0.0053 at 6% and 9% of alcohol, respectively. **d** Alcohol preference ratio was determined in GGLP1 and GWT mice. Bars represent mean ± S.E.M. (*n* = 8 per group; treatment × days interaction by repeated-measures ANOVA: *F*_2,28_ = 6.464, *P* = 0.0049). Significance was tested by Fisher’s LSD test. *P = 0.0107 and **P = 0.0062 at 6% and 9% of alcohol, respectively. **e** Saccharin solution consumption (ml) in GGLP1 and GWT mice were measured at various saccharin concentrations (*n* = 8 per group; treatment × days interaction by repeated-measures ANOVA: *F*_2,28_ = 0.1318, *P* = 0.8771). **f** Saccharin preference ratio was determined in GGLP1 and GWT mice. (*n* = 8 per group; treatment × days interaction by repeated-measures ANOVA: *F*_2,28_ = 0.0569, *P* = 0.9448).

GGLP1 and mock-grafted (GWT) mice were fed with a doxycycline diet. After 4 days of training with i.p. ethanol (1 g/kg), GWT mice stayed mostly in the chambers that had been paired with alcohol, while GGLP1 mice exhibited no such significant preference (Fig. 1a). This result suggests that grafting GLP1-producing cells reduces the development of alcohol-taking behavior. Relapse to alcohol use after withdrawal is a common feature of alcoholism. Reinstatement of alcohol CPP after extinction can be used as an experimental model to study relapse to alcohol-seeking [27, 31]. In the absence of doxycycline, we trained both GGLP1 and GWT mice to acquire CPP using alcohol (Fig. 1b, 1 g/kg). We then performed extinction training and fed the mice with a doxycycline diet 3 days prior to alcohol-elicited reinstatement (Fig. 1b). Following a priming i.p. alcohol injection (0.5 g/kg), GWT mice but not GGLP1 mice exhibited a preference for the alcohol-paired environment (Fig. 1b), indicating that GLP1 produced by the skin grafts can prevent the reinstatement of drug-seeking induced by alcohol.

We next examined the effects of skin-derived GLP1 on active alcohol drinking and preference using a two-bottle choice procedure, in which mice can choose either water or increasing doses of alcohol (3, 6, and 9%). We first established no preference at 0% for the two bottles for a week and a preference for alcohol in both GGLP1 and GWT mice by allowing mice to drink at 3% alcohol for a week. In the absence of doxycycline, both mice developed a preference for 3% alcohol and there was no difference in ethanol consumption and preference between the GGLP1 and GWT mice (Fig. 1c, d). We then increased alcohol concentration to 6% and at the same time, turned on GLP1 expression from the grafted skin by feeding mice with doxycycline-containing food. In the presence of dox, as skin-derived GLP1 started to accumulate in the blood, GGLP1 mice consumed less alcohol (g/kg of body weight per day) (Fig. 1c) and showed a lower preference ratio (volume of alcohol consumption divided by the volume of total fluid consumption) (Fig. 1d) than GWT mice at the 6% ethanol concentration. The difference in ethanol consumption and preference ratio between the two mouse groups was significant at the 9% ethanol concentration (Fig. 1c, d). Therefore, within the same group of GGLP1 and GWT mice, in the absence of dox, mice developed a similar preference for alcohol, and in the presence of dox, GGLP1 mice showed a change from preferring alcohol to a decrease in alcohol preference because of GLP1 expression from the skin, while GWT mice continue to consume more alcohol with increasing concentrations of alcohol. There were no significant differences in saccharin consumption (Fig. 1e) and preference (Fig. 1f) tested by a similar two-bottle choice protocol between GGLP1 and GWT mice, suggesting that decreased alcohol consumption and preference in GGLP1 mice is specific for alcohol.

### Cutaneous gene delivery of GLP1 decreases alcohol-induced DA accumulation in the NAc

**Fig. 2:**
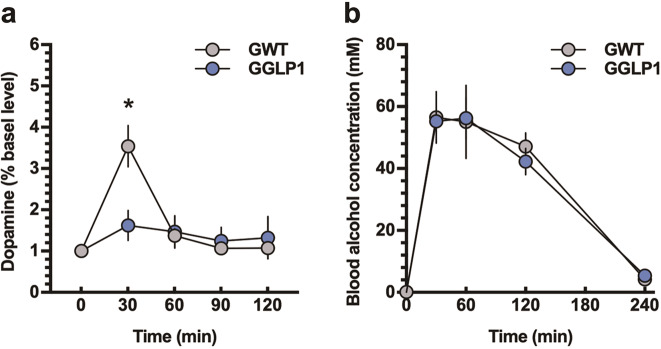
Cutaneous gene delivery of GLP1 decreases alcohol-induced DA accumulation in the NAc. **a** DA levels in the NAc following i.p. injections of 1 g/kg alcohol in GGLP1 and GWT mice (*n* = 5 per group; treatment × time interaction: *F*_4,32_ = 7.928, *P* = 0.0001 by repeated-measures ANOVA). Significance was tested by Fisher’s LSD test. *P = 0.0161 between GLP1 and GWT mice 30 min after injection. **b** Alcohol concentration in plasma. This was quantified by using ELISA following acute i.p. injections of 1 g/kg alcohol in GGLP1 and GWT mice (*n* = 6 per group; treatment × time interaction: *F*_4,40_ = 0.1178, *P* = 0.9754 by repeated-measures ANOVA).

Microdialysis in rodents indicates that alcohol enhances DA release predominantly in the NAc that is necessary for the reinforcing effects of drugs of abuse [32]. We examined whether skin-derived GLP1 has an effect on alcohol-induced elevation of DA levels in the NAc. Following an acute alcohol injection (1 g/kg) we collected dialysates from both GGLP1 and GWT mice and determined DA levels using the LC–MS method [27]. GGLP1 mice exhibited much lower extracellular DA levels in the NAc than those in GWT mice (Fig. 2a). The alcohol concentration in the blood in GGLP1 mice was similar to that in GWT mice as revealed by ELISA analysis (Fig. 2b), indicating that skin-derived GLP1 diminished DA accumulation in response to alcohol via acting in the brain but not alcohol metabolism in the peripheral.

### Development of a novel co-grafting procedure for both hBChE and GLP1 epidermal stem cells

**Fig. 3:**
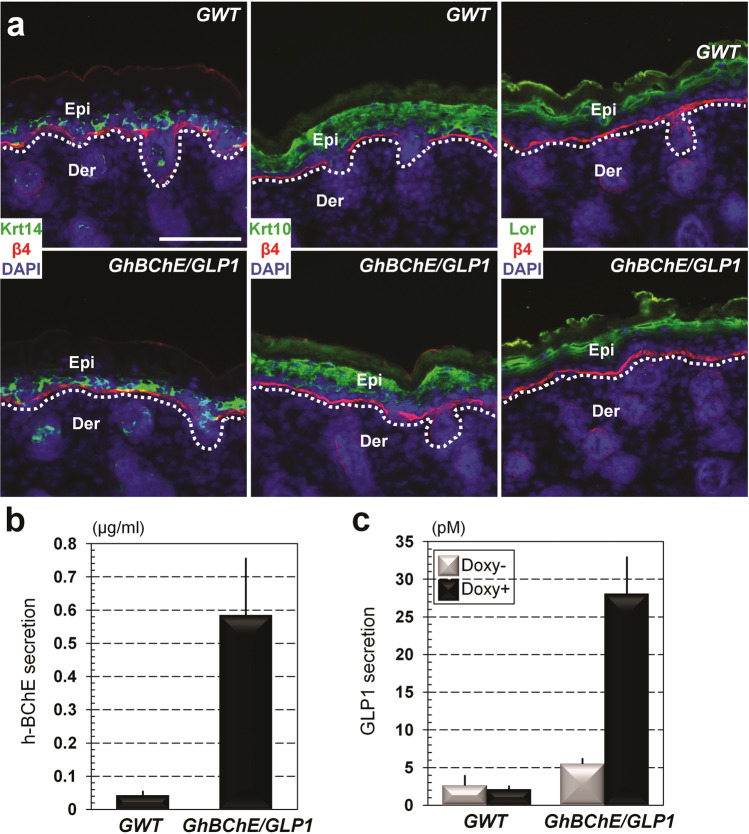
Development of a novel co-grafting procedure for both hBChE and GLP1 epidermal stem cells. **a** hBChE and GLP1 co-grafted skins. Sections of grafted skin were immunostained with various antibodies and were visualized. (Krt14: keratin 14 is a basal stem cell marker for skin epidermis; Krt10: keratin 10 is a marker for early epidermal differentiation, Lor: Loricrin is a marker for early epidermal differentiation, β4: β4-integrin, CD104 is a marker for skin basement membrane). Dashed lines represent the skin basement. Epi epidermis, Der dermis. Scale bar = 50 μm. **b**, **c** Levels of hBChE and GLP1 in the co-grafted mice. The presence of hBChE (**b**) and GLP1 (**c**) in blood was quantified by ELISA (*n* = 3 mice per group). *GLP1* expression was induced by doxycycline (Dox) diet in GhBChE/GLP1 mice.

The simultaneous use of hBChE and GLP1 may allow more efficient treatment of alcohol and cocaine co-abuse because hBChE can efficiently degrade cocaine and cocaethylene thereby reducing their rewarding and toxic effects while GLP1 can attenuate reinforcing properties induced by both alcohol and cocaine. In addition to skin stem cells carrying the doxycycline-inducible GLP1 gene, we previously also made skin stem cells containing the hBChE gene under the control of a constitutive ubiquitin C promoter [27]. The engineered skin grafts produced high levels of hBChE in vivo which were very effective in clearing cocaine, decreasing DA levels in the brain, and protecting mice from developing cocaine taking, seeking, and toxicity [27]. To evaluate the effectiveness of the skin cell delivery platform in reducing concurrent alcohol and cocaine use, we developed a new organotypic culture model in vitro by coculturing the hBChE and GLP1*-*expressing skin stem cells on mouse dermis [26, 33]. Similar to individual cultures for hBChE and GLP1, we were able to generate a skin-like organoid cell culture. Following transplantation to WT recipient mice (Fig. 3a), there was no significant rejection of the skin grafts, indicating that the engineered skin cells were immunologically tolerable by the hosts. Grafted skin cells with hBChE and GLP1 expression exhibited normal epidermal differentiation and tissue architecture as control cells (Fig. 3a). Mice that were co-grafted with hBChE and GLP1*-*producing cells showed significantly higher expression of hBChE and GLP1 in plasma (Fig. 3b, c). Consistent with previous reports [34], therapeutic peptides and proteins expressed in the skin epidermis were able to enter the blood circulation in vivo.

### Epidermal stem cell-derived hBChE and GLP1 protect mice from reward and toxicity induced by co-administration of alcohol and cocaine

**Fig. 4:**
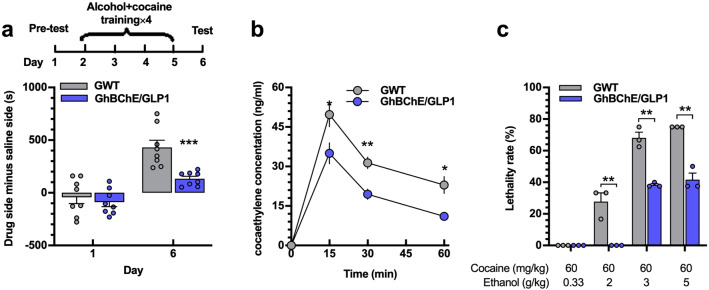
Epidermal stem cell-derived hBChE and GLP1 protect mice from reward and toxicity induced by co-administration of alcohol and cocaine. **a** GhBChE/GLP1 and GWT mice were fed with doxycycline and underwent pre-test, alcohol (1 g/kg) and cocaine (10 mg/kg) conditioning and expression test. Data were plotted as means ± S.E.M. (*n* = 8 mice per group; treatment × days interaction by repeated-measures ANOVA: *F*_1,14_ = 13.46, *P* = 0.0025). Significance was tested by Fisher’s LSD test. ***P = 0.0003 on Day 6. **b** Cocaethylene concentration in the blood was determined by LC–MS/MS after cocaine and alcohol administration in GhBChE/GLP1 and GWT mice (*n* = 5 for each group; treatment × time interaction: *F*_3,24_ = 4.6, *P* = 0.0111, repeated-measures two-way ANOVA). Significance was tested with Fisher’s LSD test. *P = 0.0414, **P = 0.0035, and *P = 0.0165 at 15, 30, and 60 min, respectively. **c** Lethality rates after injection of multiple doses of cocaine-alcohol in GhBChE/GLP1 and GWT mice. Bars show means ± S.E.M. (Three independent experiments were performed and 6–8 mice were used for each experiment). Individual data points show the result from each experiment. Significance was tested by Student’s *t* test. *t* = 5.018, **P = 0.0074 at 60 mg/kg cocaine plus 2 g/kg ethanol injections; *t* = 7.895, **P = 0.0014 at 60 mg/kg cocaine plus 3 g/kg ethanol injections; *t* = 8, **P = 0.0013 at 60 mg/kg cocaine plus 5 g/kg ethanol injections.

GhBChE/GLP1 and GWT mice were fed with a doxycycline diet and conditioned with sequential injections of alcohol (1 g/kg) and cocaine (10 mg/kg). Following 4 days of training, GhBChE/GLP1 mice stayed much less in chambers where they received cocaine and alcohol co-administration than that of GWT mice (Fig. 4a). This result implicates that skin graft-derived hBChE and GLP1 decrease the place preference induced by cocaine and alcohol co-injections. Cocaethylene is the major intermediate that is generated by cocaine and alcohol co-administration, producing a strong reinforcing effect and cardiotoxicity. We measured the plasma cocaethylene concentration in GhBChE/GLP1 and GWT mice. After an acute injection of both cocaine and alcohol (60 mg/kg cocaine plus 0.33 g/kg ethanol), GhBChE/GLP1 mice exhibited a much faster cocaethylene clearance in the NAc than that in GWT mice (Fig. 4b). Thus, co-delivery of hBChE and GLP1 via epidermal stem cell can decrease reward induced by cocaine and alcohol co-administration and clear cocaethylene at a faster rate.

To determine whether the co-delivery of hBChE and GLP1 protects mice from acute toxicity of co-exposure to cocaine and alcohol, we injected a constant dose of cocaine and increasing doses of alcohol using different groups of GhBChE/GLP1 and GWT mice and recorded the number of deaths. Totally, 60 mg/kg cocaine plus 0.33 g/kg ethanol produced no deaths in GhBChE/GLP1 and GWT mice (Fig. 4c). By contrast, 60 mg/kg cocaine plus 2 g/kg ethanol had nearly no lethality in GhBChE/GLP1 mice but produced 27.5% lethality in GWT mice, 60 mg/kg cocaine plus 3 g/kg ethanol produced 38.3% lethality in GhBChE/GLP1 mice and 68% lethality in GWT mice, and 60 mg/kg cocaine plus 5 g/kg ethanol produced 41.6% lethality in GhBChE/GLP1 mice and 75% lethality in GWT mice (Fig. 4c). This result indicates that grafting both hBChE and GLP1-producing cells significantly reduces lethality induced by multiple doses of cocaine and alcohol co-administration.

## Discussion

Our current study demonstrates that a GLP1 gene delivered via genomic engineered skin graft can reduce alcohol-induced acquisition of drug-taking and reinstatement of drug-seeking as well as ongoing alcohol drinking. Using a newly developed co-grafting strategy, we show that that co-delivery of hBCHE and GLP1 genes can decrease reward and toxicity induced by cocaine and alcohol co-administration. These results imply that cutaneous gene delivery through skin transplants may add a new option to treat drug abuse and co-abuse.

For reducing effects of alcohol, we chose GLP1 as the intervening agent because (1) many previous pharmacological studies show that GLP1 acting through GLP1 receptors can reduce the reinforcing effects of alcohol, cocaine, and other addictive drugs [19, 20, 35]; (2) GLP1 is an endogenous peptide that can gain access to the brain from the periphery by simple diffusion to exert central effects [36]. To be able to reversibly regulate its expression in skin cells, we used the doxycycline-dependent promoter to control GLP1 expression. This design allowed us to study the effects of skin-derived GLP1 on multiple aspects of alcohol abuse including acquisition, reinstatement, and ongoing drinking. Doxycycline is a widely used antibiotic in humans [37], implying a possible clinical use of controllable GLP1 gene therapy in the future. To explore the potential of the skin cell-based gene delivery platform on drug abuse, we used the CPP paradigm which is widely thought to measure the motivational effects of drugs of abuse, and two-bottle choice to determine the extent of oral alcohol self-administered, which is an ideal model of alcohol drinking and taking in AUD patients. Our results show that GLP1 delivery in the blood can inhibit different aspects of alcohol-induced reward behaviors (Fig. 1). It is thought that the basal lateral amygdala, the NAc, and related projections are closely related to the acquisition of drug-induced CPP including that induced by alcohol [38]. Multiple brain regions including the amygdala, prefrontal cortex, hippocampus, and bed nucleus of the stria terminalis and related circuits are implicated in the reinstatement of conditioned responses to drugs of abuse [39]. The mesolimbic DA system projects to all these brain regions. Since GLP1 and GLP1R agonists can inhibit the activity of the mesolimbic system [19, 20] and DA accumulation in the NAc in the brain (Fig. 2), it is thus possible that GLP1 reduces acquisition of alcohol-taking and reinstatement of alcohol-seeking by reducing central DA actions. These results suggest that cutaneous gene delivery through a single skin transplant may add a new tool to treat drug abuse.

For reducing effects induced by the co-exposure of alcohol and cocaine, we used both hBChE and GLP1. The simultaneous use of hBChE and GLP1 may provide added assurance for reducing cocaine abuse because hBChE can efficiently degrade cocaine [27] and clear cocaethylene thereby reducing their rewarding and toxic effects while GLP1 can attenuate reinforcing properties induced by both cocaine and alcohol (Fig. 1) [15, 17]. For this reason, we developed a coculture and co-grafting (Fig. 3a) procedure that allowed simultaneous delivery and expression of both hBChE and GLP1 in GhBChE/GLP1 mice (Fig. 3b, c). Coculture allows adjusting ratios of the hBChE and GLP1-expressing cells to eventually produce desired gene expression in grafted mice. The resulting GhBChE/GLP1 mice show attenuated reward behaviors induced by cocaine and alcohol co-administration (Fig. 4a). Moreover, cocaethylene was cleared at a faster rate in GhBChE/GLP1 mice than in GWT mice (Fig. 4b), contributing to the attenuated acquisition of reward (Fig. 4a) and also resulting in significantly reduced lethality induced by these two drugs (Fig. 4c). Whereas it remains to be determined whether increasing the ratio of hBChE to GLP1 cells co-grafted would improve on protection against the lethality induced by cocaine and alcohol co-injections, the current results imply that cutaneous gene delivery through skin transplants may provide a therapeutic strategy to treat co-abuse of different combinations of drugs.

Our findings described in the current study provide a basis for additional future studies. The genetically modified skin graft is capable of stably expressing GLP1 for at least 16 weeks in the continuous presence of dox [26]. We have not systematically tested how much longer beyond the 16 weeks this platform remains effective in GGLP1 mice. Human grafts used in treating burn patients can last for a lifetime [40]. In addition, a recent study demonstrated that cultured epidermal autografts could be used to replace the entire skin epidermis, and this procedure saved the life of a patient from junctional epidermolysis bullosa which is a potentially life-threatening skin disease [41]. Together, they strongly support the long-term feasibility of cutaneous gene therapy. We know the hBChE skin grafts last for at least 7 months (unpublished). As such, we believe the GLP1 skin graft will last much longer than 16 weeks in mice and we will systematically address this issue for GLP1, hBChE, and GLP1-hBChE co-grafts in the future.

It is yet unknown that whether this gene delivery platform can reduce reinstatement induced by drug-associated cues or stress. Exendin-4, a GLP1R agonist, can reduce cue-induced heroin-seeking in a self-administration model [42]. Whether skin-derived GLP1 can reduce cue-elicited drug-seeking needs to be addressed in the future. Moreover, previous studies show that GLP1 in the brain can activate stress response [43], and the central GLP1 system is activated in response to stress [44, 45]. It will be interesting to investigate the role of GLP1 in stress-induced drug-seeking. Although many studies demonstrated that stimulation of GLP1R inhibits the rewarding effects of alcohol, cocaine, and nicotine [15, 35, 46], the neuronal circuits responsible for this inhibitory effect is not fully elucidated. It is reported that GLP1 can stimulate medial habenular (MHb) projections to the interpeduncular nucleus (IPN) and activates this circuit decreases nicotine reward and intake [46]. It is known that MHb and IPN circuitry is also critical in the development of addiction to alcohol, opioid, and stimulants [47]. Thus, it is possible that GLP1 inhibits alcohol, cocaine and opioid rewards via activation of GLP1 neurons in a shared MHb-IPN pathway. Repeated use of drugs of abuse including alcohol and cocaine has been documented to produce numerous neuroadaptive changes. How repeated exposure to alcohol and/or cocaine impacts endogenous GLP1 function needs to be explored. Despite these unresolved issues, our epidermal stem cell-based genetic platform is likely long-lasting, highly efficient with little individual variation, minimally invasive, has the potential to be safe with minimal immune responses, and low maintenance. This platform offers a valuable vehicle to deliver one or multiple genes as therapeutic agents in the context of alcohol and/or cocaine co-abuse specifically, and drug abuse and co-abuse in general.

## Data Availability

The data that support the present study are available within the paper. Original data from Fig. 1, Fig. 2, and Fig. 4 have been placed in Figshare repository https://figshare.com/articles/dataset/source_data_xlsx/13169549.
